# Supplementary material for: Immune Sensitization to Mycobacterium tuberculosis Among Young Children with and without Tuberculosis
Source: Pathogens. 2025 Sep 12;14(9):924. doi: 10.3390/pathogens14090924 (PMC12472572; doi:10.3390/pathogens14090924)
Supplement: Supplementary file 1 [file pathogens-14-00924-s001.zip › pathogens-3819971-supplementary.pdf]

SUPPLEMENTARY FILE

Supplemental Table 1: Presence of symptoms and symptom duration of the index cases of study participants with and without TB

Symptoms/Symptom duration (days)	Index of full cohort	Index of PedAS	Index of PedTB	p-value (test)
Cough = present	130 (100)	75 (100)	55 (100)	NA
Cough duration	60 [30-150]	60 [30-180]	60 [30-120]	0.98 (MWU)
Fever = present	76 (65)	43 (63.2)	33 (67.3)	0.66 (χ^2)
Fever duration	30 [21-60]	30 [21-60]	30 [30-150]	0.12 (MWU)
Productive sputum = present	114 (97.4)	68 (100)	46 (93.9)	0.14 (χ^2)
Productive sputum duration	60 [30-112.5]	60 [30-97.5]	60 [30-112.5]	0.84 (MWU)
Purulent sputum = present	91 (77.8)	56 (82.4)	35 (71.4)	0.24 (χ^2)
Purulent sputum duration	60 [30-90]	60 [30-97.5]	30 [25.5-90]	0.25 (MWU)
Night sweats = present	76 (65)	39 (57.4)	37 (75.5)	0.07 (χ^2)
Night sweats duration	52.5 [21-90]	60 [30-90]	45 [21-90]	0.94 (MWU)
Hemoptysis = present	14 (12)	7 (10.3)	7 (14.3)	0.71 (χ^2)
Hemoptysis duration	6 [3-21]	21 [4.5-21]	3 [3-6]	0.44 (MWU)
Weight loss = present	99 (84.6)	57 (83.8)	42 (85.7)	0.98 (χ^2)
Weight loss duration	60 [30-105]	60 [30-120]	45 [30-90]	0.57 (MWU)

Counts (percentages) or median [quartiles]
PedAS: Not diagnosed with TB
PedTB: Diagnosed with TB
χ^2: Chi-squared test
MWU: Mann–Whitney *U* test

Supplemental Table 2: Final diagnostic criteria for Asymptomatic Mtb-exposure and tuberculosis disease (TB)

Asymptomatic Mtb-exposure
Child has a known TB household exposure, and:
<u>At enrolment were negative for:</u>
- TB signs and symptoms

AND
- CXR findings consistent with TB disease

AND
- AFB culture and GeneXpert sputum test results

AND
- Not treated for TB disease during the 12 month study period

AND
- Remained asymptomatic for TB during the 12 month study period

Unconfirmed TB
Child has a known TB household exposure, plus:
<u>At least one of the following:</u>
- Symptoms/signs suggestive of tuberculosis: cough ≥ 14 days, fever ≥ 7 days, poor appetite, known weight loss or failure to thrive, fatigue and/or reduced playfulness

AND/OR
- Chest x-ray findings consistent with pulmonary TB

AND/OR
- Documented positive response to TB treatment

AND
- Negative microbiological testing

Confirmed TB
Positive microbiological testing with or without positive signs and symptoms and chest x-ray consistent with pulmonary TB

Supplemental Table 3: Nutritional status among children with and without TB at study entry

Z Scores	PedAS	PedTB	p-value (test)
n	78	54	
Weight	-0.72 [+/- 1.18]	-0.41 [+/- 1.18]	0.14 (t-test)
Length/Height	-0.92 [+/- 1.56]	-0.72 [+/- 1.24]	0.41 (t-test)
Weight for Length/Height	-0.29 [+/- 1.28]	-0.03 [+/- 1.10]	0.21 (t-test)
BMI	-0.21 [+/- 1.31]	0.03 [+/- 1.14]	0.26 (t-test)
Upper arm Circumference	-0.54 [+/- 1.03]	-0.36 [+/- 0.98]	0.32 (t-test)

Means [+/- standard deviation]
PedAS: Not diagnosed with TB
PedTB: Diagnosed with TB
BMI: Body mass index

Supplemental Table 4: Cross-tabulation of QFT-Plus and TST results (10 mm cutoff) for PedAS (Not diagnosed with TB) participants

		TST Results (10 mm cutoff)			
PedAS	QFT-Plus Results	**N=73***	**Positive**	**Negative**	
		**Positive**	19 (26%)	2 (2.7%)	McNemar χ^2 = 0.68
		**Negative**	4 (5.5%)	48 (65.8%)	Cohen's Kappa[a] = 0.80
Children <2 years of age	QFT-Plus Results	**N=33**	**Positive**	**Negative**	
		**Positive**	8 (24.3%)	0 (0%)	McNemar χ^2 = 0.13
		**Negative**	4 (12.1%)	21 (63.6%)	Cohen's Kappa[a] = 0.72
Children 2-5 years of age	QFT-Plus Results	**N=40**	**Positive**	**Negative**	
		**Positive**	11 (27.5%)	2 (5%)	McNemar χ^2 = 0.48
		**Negative**	0 (0%)	27 (67.5%)	Cohen's Kappa[a] = 0.88

*Does not include 2 participants with indeterminate QFT results

[a]Cohen's Kappa interpretation: 0.01–0.20 as none to slight, 0.21–0.40 as fair, 0.41– 0.60 as moderate, 0.61–0.80 as substantial, and 0.81–1.00 as almost perfect agreement.

Supplemental Table 5: Cross-tabulation of QFT-Plus and TST results (10 mm cutoff) for PedTB (Diagnosed with TB) participants

		TST Results (10 mm cutoff)			
**PedTB**	**QFT-Plus Results**	**N=52***	**Positive**	**Negative**	
		**Positive**	18 (34.6%)	2 (3.8%)	McNemar χ^2 = 0.18
		**Negative**	7 (13.5%)	25 (48.1%)	Cohen's Kappa[a] = 0.65
**Children <2 years of age**	**QFT-Plus Results**	**N=20**	**Positive**	**Negative**	
		**Positive**	3 (15%)	1 (5%)	McNemar χ^2 = 0.37
		**Negative**	4 (20%)	12 (60%)	Cohen's Kappa[a] = 0.39
**Children 2-5 years of age**	**QFT-Plus Results**	**N=32**	**Positive**	**Negative**	
		**Positive**	15 (46.9%)	1 (3.1 %)	McNemar χ^2 = 0.62
		**Negative**	3 (9.4%)	13 (40.6%)	Cohen's Kappa[a] = 0.75

*Does not include 3 participants with indeterminate QFT results

[a]Cohen's Kappa interpretation: 0.01–0.20 as none to slight, 0.21–0.40 as fair, 0.41– 0.60 as moderate, 0.61–0.80 as substantial, and 0.81–1.00 as almost perfect agreement.

Supplemental Table 6: Cross-tabulation of QFT and TST results (5 mm cutoff) for PedAS (Not diagnosed with TB) participants

		TST Results (5 mm cutoff)			
**PedAS**	**QFT-Plus Results**	**N=73***	**Positive**	**Negative**	
		**Positive**	20 (27.4%)	1 (1.4%)	McNemar $\chi^2 = 0.03$
		**Negative**	9 (12.3%)	43 (58.9%)	Cohen's Kappa[a] = 0.70
**Children <2 years of age**	**QFT-Plus Results**	**N=33**	**Positive**	**Negative**	
		**Positive**	8 (24.2%)	0 (0%)	McNemar $\chi^2 = 0.02$
		**Negative**	7 (21.2%)	18 (54.6%)	Cohen's Kappa[a] = 0.55
**Children 2-5 years of age**	**QFT-Plus Results**	**N=40**	**Positive**	**Negative**	
		**Positive**	12 (30%)	1 (2.5%)	McNemar $\chi^2 = 1$
		**Negative**	2 (5%)	25 (62.5%)	Cohen's Kappa[a] = 0.83

*Does not include 2 participants with indeterminate QFT results
[a]Cohen's Kappa interpretation: 0.01–0.20 as none to slight, 0.21–0.40 as fair, 0.41– 0.60 as moderate, 0.61–0.80 as substantial, and 0.81–1.00 as almost perfect agreement.

Supplemental Table 7: Cross-tabulation of QFT and TST results (5 mm cutoff) for PedTB (Diagnosed with TB) participants

		TST Results (5 mm cutoff)			
PedTB	QFT-Plus Results	**N=52***	**Positive**	**Negative**	
		**Positive**	18 (34.6%)	2 (3.9%)	McNemar $\chi^2 = 0.01$
		**Negative**	13 (25%)	19 (36.5%)	Cohen's Kappa[a] = 0.45
Children <2 years of age	QFT-Plus Results	**N=20**	**Positive**	**Negative**	
		**Positive**	3 (15%)	1 (5%)	McNemar $\chi^2 = 0.08$
		**Negative**	7 (35%)	9 (45%)	Cohen's Kappa[a] = 0.20
Children 2-5 years of age	QFT-Plus Results	**N=32**	**Positive**	**Negative**	
		**Positive**	15 (46.9%)	1 (3%)	McNemar $\chi^2 = 0.13$
		**Negative**	6 (18.8%)	10 (31.3%)	Cohen's Kappa[a] = 0.56

*Does not include 3 participants with indeterminate QFT results
[a]Cohen's Kappa interpretation: 0.01–0.20 as none to slight, 0.21–0.40 as fair, 0.41– 0.60 as moderate, 0.61–0.80 as substantial, and 0.81–1.00 as almost perfect agreement.

Supplemental Table 8: Logistic regression model of predictors of PedTB (Diagnosed with TB) adjusted for age (months), sex, HIV status, and BCG status

COVARIATES	ADJUSTED OR	95% CI	p-VALUE
Quantitative TST result (in mm)	1.04	1.001 – 1.09	0.051*
Sex = male	0.90	0.42 – 1.91	0.78
Age (months)	1.01	0.99 – 1.04	0.21
HIV = positive	0.91	0.04 – 10.42	0.94
BCG scar = present	1.25	0.44 – 3.77	0.68

*** Statistically significant at p<0.05**
CI: Confidence interval

Supplemental Table 9: Quantitative QFT-Plus results summary (n=125*) by Tube

	Minimum	First quartile	Median	Third quartile	Maximum	Mean	Standard deviation
**Tube TB.1**	0	0	0.01	1.85	10	1.94	3.39
**Tube TB.2**	0	0	0.02	2.78	10	1.99	3.38

*Does not include five participants with indeterminate results

Supplemental Table 10: Quantitative IGRA results among 3 participants with discordant QFT-Plus Tube TB.1 minus nil and Tube TB.2 minus mil results

Age group	Tube TB.1	Tube TB.2
Older than 2 years	0.02 IU	3.01 IU
Older than 2 years	0.34 IU	0.55 IU
Older than 2 years	0.97 IU	0.00 IU

Supplemental Figure 1: Form used for the evaluation of chest x-rays obtained during the study

32818

UGANDA CWRU RESEARCH COLLABORATION

Adaptive Immunity to MTB Infection and Disease

Chest X-ray Evaluation Form

DATA QUALITY CONTROL

Note: Two readings on separate CRFs required for PA21 Study (AP and lateral)

Clinical Coordinator 24-hour Review — CC Initials ☐☐ Date ☐☐ / ☐☐ / 2 0 ☐☐

Data Management Key Variable Review — DM Initials ☐☐ Date ☐☐ / ☐☐ / 2 0 ☐☐

1. IDNO ☐☐☐☐☐ - ☐ - ☐☐

2. TB Control Program IDNO ☐

3. Date of Chest X-Ray ☐☐ / ☐☐ / ☐☐☐☐ (Day / Month / Year)

4. Review of:
- ☐ Anterior-Posterior
- ☐ Lateral

5. Is X-Ray adequate?
- ☐ Yes
- ☐ No

6. Visit
- ☐ P1
- ☐ P8

Initials (Site use only) ☐☐☐ (First, Middle, Last)

7. Extent of Lung Disease on Chest X-Ray ☐

0 = Normal
1 = Minimal disease -- infiltrates of slight to moderate density; disease may be present in a small portion of both lungs; the total volume of the infiltrate(s) must be the volume of one lung present above the second chondrosternal junction and the spine of the fourth or the body of the fifth thoracic vertebra; no cavitation may be present.
2 = Moderately advanced disease -- disease may be present in one or both lungs; the total extent must not be more than the following:
(a) scattered lesions of slight to moderate density may not involve more than total volume of one lung or the equivalent volume of both lungs.
(b) dense, confluent lesions may not involve more than 1/3 of the volume of one lung.
(c) the total diameter of cavity(ies) may not be greater than 4 cm.
3 = Far advanced -- lesions more extensive than moderately advanced.

8. Disease present in ☐ 1 = Right chest ☐ 2 = Left chest ☐ 3 = Both

9. Number of Lung Zones involved by Disease (out of 6) ☐

SPECIFIC RADIOGRAPHIC FINDINGS

	BASELINE evaluation only: 1=Present 0=Absent	Follow-up evaluation only: N = New, S = Same, B = Better, W=Worse, R = Resolved (since last x-ray), A = Absent (not found this or prior x-ray)	Significant Interval Change: 1 = Yes, 0 = No, 9 = Uncertain
**10. Infiltrates:** *(i.e. above & below a line drawn between pulmonary hila)*			
a. Upper Lung Field	☐	☐	☐
b. Lower Lung Field	☐	☐	☐
11. Fibrosis / volume loss	☐	☐	☐
12. Cavity(ies)....................	☐	☐	☐
13. Miliary Disease...........	☐	☐	☐
14. Adenopathy..................	☐	☐	☐
15. Pleural Effusion...........	☐	☐	☐
16. Pleural Thickening......	☐	☐	☐
17. Other............................	☐	☐	☐

18. If other, specify: ____________

19. Grade Overall Changes in most recent Chest X-Ray ☐

0 = Baseline film, no comparison
1 = Improved
2 = Same
3 = Worse
9 = Prestudy film

20. Comments: ____________

21. Read by (Initials) ☐☐ (First name, Last name)

22. Date ☐☐ / ☐☐ / 2 0 ☐☐ (Day / Month / Year)

PA21 Chest X-Ray CRF (page 1 of 1)
Version Date: Nov 2021

Medical Examiner Signature and credentials ____________
